# Tourette syndrome and chronic tic disorder are associated with lower socio-economic status: findings from the Avon Longitudinal Study of Parents and Children cohort

**DOI:** 10.1111/dmcn.12318

**Published:** 2013-10-19

**Authors:** Laura L Miller, Jeremiah M Scharf, Carol A Mathews, Yoav Ben-Shlomo

**Affiliations:** 1 School of Social and Community Medicine, University of BristolBristol, UK; 2 Psychiatric and Neurodevelopmental Genetics Unit Center for Human Genetics Research Departments of Neurology and Psychiatry, Massachusetts General HospitalBoston, MA, USA; 3 Division of Cognitive and Behavioral Neurology Department of Neurology, Brigham and Women’s HospitalBoston, MA, USA; 4 Department of Psychiatry, University of CaliforniaSan Francisco, CA, USA

## Abstract

**Aim** Only a few studies have examined the relationship between Tourette syndrome or chronic tic disorder and socio-economic status (SES). Existing studies are primarily cross-sectional, arise from specialty clinics, and use single measures of SES. In this study we examine this relationship in a longitudinal, population-based sample.

**Method** Data are from 7152 children born during 1991 and 1992 in the county of Avon, UK, from the Avon Longitudinal Study of Parents and Children, who were followed up to age 13. After exclusions for intellectual disability* and autism, 6768 participants (3351 males [49.5%]) and 3417 females [50.5%]) remained. Parental SES was assessed using multiple measures during pregnancy and at 33 months of age. Presence of Tourette syndrome or chronic tics was determined from repeated maternal questionnaires up to when the child was 13 years of age.

**Results** Multiple SES measures were associated with an approximately twofold increased risk of Tourette syndrome and chronic tics. A postnatal composite factor score (lowest vs highest tertile odds ratio 2.09, 95% confidence interval 1.38–3.47) provided the best fit to the data.

**Interpretations** As is seen in several childhood conditions, such as cerebral palsy and autism, lower SES is a risk factor for Tourette syndrome/chronic tics. Potential explanations include differential exposure to environmental risk factors or parental psychopathology as a measure of an increased genetic risk leading to decreased parental SES.

Tourette syndrome is a chronic neuropsychiatric disorder that starts in childhood and is characterized by motor and vocal tics persisting for more than 1 year and varying in frequency.^1^ Chronic motor or vocal tic disorder is defined by the presence of either motor or vocal tics (but not both) and shares a similar clinical phenomenology and disease course with Tourette syndrome. Tourette syndrome and chronic tics can cause appreciable physical and psychosocial impairment, and in severe cases can result in lifelong disability.^2^,^3^ Remarkably little is known about the aetiology of Tourette syndrome and chronic tics, although it is presumed that both genetic and environmental factors are likely to play a role in their development.^4^ The examination of disease patterning by socio-economic status (SES) may provide insight into the aetiology of Tourette syndrome and chronic tics as it has for other disorders, owing to the social patterning of risk factors. For example, the strong association between childhood socio-economic conditions and stomach cancer mortality, independent of adult status, suggests the potential role of *Helicobacter pylori* infection as an aetiological factor given its association with overcrowding in childhood.^5^

The majority of studies have suggested that there is no increased risk of Tourette syndrome and chronic tics among lower SES groups.^6^–^8^ These studies have mostly been conducted in clinical samples, leading to a potential ascertainment bias, as individuals of lower SES may be less likely to seek or obtain treatment, even in universal health care systems.^9^ In contrast, two published population-based cross-sectional studies found that tics were either not associated with SES^10^ or were associated with lower SES,^11^ although the former study was underpowered (based on only 24–34 individuals), and both studies used simple measures of SES.

In the present study, we report the relationship between a comprehensive range of SES measures and Tourette syndrome/chronic tics from the Avon Longitudinal Study of Parents and Children (ALSPAC) study, an ongoing, prospective, population-based birth cohort study. We have thus eliminated recall bias and reverse causation and reduced ascertainment bias compared with case–control studies. The aims were to determine (1) whether lower SES was associated with an increased risk of Tourette syndrome and chronic tics in a population unselected for the presence of tic disorders, and (2) if the use of a composite measure of SES was a better indicator of risk than a single measure.

## Method

### Participants

The ALSPAC recruited 14 541 pregnant females resident in the former county of Avon, UK, with expected delivery dates between 1 April 1991 and 31 December 1992 (85% of the eligible population).^12^ This resulted in 14 676 fetuses, of whom 14 062 were live births and 13 988 were alive at 1 year of age. Mothers completed self-administered questionnaires about themselves and their child’s development, environmental exposures, and health outcomes during pregnancy approximately every 6 months from birth to 7 years of age and every year thereafter, with data available for 7152 children at 13 years of age (age at the administration of the detailed tic questionnaire). Ethical approval for the study was obtained from the ALSPAC law and ethics committee and the local research ethics committees.

### Disease definitions

A full description and definitions of Tourette syndrome and chronic tic disease derived from the ALSPAC cohort and their validity can be found elsewhere.^13^ Briefly, diagnoses of Tourette syndrome and chronic tics were made after DSM-IV^1^ (Text Revision) criteria were met. Diagnosis required the presence of multiple motor and/or vocal tics based on five tic-related questions in the maternal report questionnaire completed when the child was 13 years of age. Additionally, a positive response was required for at least one of the single-tic screening questions administered at eight time points when the child was between 1 year 6 months and 10 years of age (to meet the criterion of tic persistence >1y). Participants with intellectual disability or autism were excluded from the sample population before study participants and the comparison group were defined to remove individuals with perseverative behaviours and stereotypies that might result in a false-positive screening result. Tourette syndrome was examined independently, as well as jointly with chronic tics as a single variable (Tourette syndrome/chronic tics) to increase statistical power.

After exclusions for intellectual disability and autism, 6768 participants remained (3351 males [49.5%]) and 3417 females [50.5%]). Fifty children (0.7%) were identified as having Tourette syndrome based on the previously described definition and 72 children (1.1%) had chronic tics, resulting in 122 children with either Tourette syndrome or chronic tics (combined prevalence of 1.8% for Tourette syndrome/chronic tics). There were 5968 children in the comparison group, defined as children eligible for analysis at 13 years of age but who did not have evidence of tics. In total, 678 individuals with non-specific tics that did not meet Tourette syndrome or chronic tic criteria were excluded from the analyses.^13^

**What this paper adds**

- Lower SES is associated with a twofold greater risk of Tourette syndrome/chronic tics.
- This association was examined using a large prospective birth cohort (ALSPAC) and a multidimensional composite measure.
- Single or area-based measures may be inadequate.
- Genetic factors or social patterning of an adverse environmental aetiological factor may explain this observation.

### Socio-economic status and demographic variables

The following range of potential individual-level SES variables used in previous UK studies were examined: (1) educational level; (2) occupational-based social class; (3) housing tenure; (4) car ownership; (5) crowding index; (6) household-based measures (e.g. private garden/yard); and (7) self-reported financial difficulties measured either during pregnancy or at 33 months of age. Further details can be found in Appendix S1 (online supporting information).

In addition, an ecological measure of area deprivation (the index of multiple deprivation) was derived from a weighted score of seven domains (income; employment; health and disability; education, skills, and training; barriers to housing and services; crime; and living environment).^14^ The index of multiple deprivation used data from lower-layer super-output areas, the smallest geographical level of aggregation, with around 400 households and a mean population of 1500 individuals each.

### Statistical analysis

#### Descriptive analyses

We examined the relationship between SES and both Tourette syndrome and Tourette syndrome/chronic tics, assuming that Tourette syndrome and chronic tics spectra differ by severity rather than being qualitatively different.^15^ The association between SES and Tourette syndrome or Tourette syndrome/chronic tic status was calculated for each individual SES variable using *p*-values for heterogeneity or trend (for social class and overcrowding).

#### Factor analyses

We conducted three separate factor analyses (using the principal-factor method for calculating the correlation matrix) to create composite measures of SES: (1) prenatal – SES variables measured during pregnancy; (2) postnatal – SES variables assessed when the child was between the ages of 2 years and 3 years; and (3) combined –both pre- and postnatal SES variables. The education and overcrowding variables were recoded so that all variables were in the same direction (i.e. a higher factor score would relate to poorer SES). Eigenvalues were required to be above 1 and examination of the scree plots was carried out to identify the appropriate number of factors. Varimax rotation was used if more than one factor was found. Standardized factor scores were created and divided into tertiles to use as an ordinal score in the regression analyses.

#### Regression analyses

We used logistic regression models to calculate odds ratios (95% confidence intervals and *p*-values) for each SES category compared with the baseline for the standardized composite SES factor scores and for each individual SES variable found to be significantly associated with Tourette syndrome or Tourette syndrome/chronic tics. We then used the Akaike information criterion and Bayesian information criterion to determine which of the associated variables was the best fit and, therefore, the most appropriate for use in future studies of Tourette syndrome/chronic tics.

## Results

### Socio-economic measures and Tourette syndrome

Several measures of adverse social circumstances were associated with Tourette syndrome and Tourette syndrome/chronic tics (Table 1). Both perinatal and postnatal financial difficulties were more common for parents of children with Tourette syndrome or Tourette syndrome/chronic tics, although the effect was stronger and more consistent for the postnatal variables. Parents of children with Tourette syndrome and Tourette syndrome/chronic tics were also more likely to be renting their property and less likely to own a car than parents of children in the comparison group. Associations between parental education, social class, housing tenure, and overcrowding and Tourette syndrome or Tourette syndrome/chronic tics were consistent with chance.

**Table 1:** Association of socio-economic status variables with Tourette syndrome and chronic tics

Variable	Category	No Tourette syndrome or chronic tics, *n* (%)	Tourette syndrome, *n* (%)	*p*	Tourette syndrome/chronic tics, *n* (%)	*p*
Perinatal factors						
Maternal education, y	<16	1109 (19.9)	11 (23.9)	0.28	20 (17.7)	0.78
	16	1965 (35.3)	11 (23.9)		39 (34.5)	
	>16	2499 (44.8)	24 (52.2)		54 (47.8)	
Partner’s education, y	<16	1162 (21.6)	13 (31.0)	0.23	25 (23.2)	0.44
	16	1212 (22.6)	6 (14.3)		25 (26.9)	
	>16	3000 (55.8)	23 (54.8)		54 (50.0)	
Maternal grandmother’s education, y	<16	2623 (61.1)	31 (73.8)	0.06	56 (60.2)	0.14
	16	671 (15.6)	1 (2.4)		9 (9.7)	
	>16	1001 (23.3)	10 (23.8)		28 (30.1)	
Maternal grandfather’s education, y	<16	2326 (57.6)	23 (62.2)	0.53	48 (51.6)	0.26
	16	515 (12.8)	6 (16.2)		17 (18.3)	
	>16	1198 (29.7)	8 (21.6)		28 (30.1)	
Housing tenure (pregnancy)	Mortgaged	4703 (84.4)	37 (80.4)	0.46	93 (80.9)	0.30
	Rented	868 (15.6)	9 (19.6)		22 (19.1)	
Social class	I/II	3351 (62.9)	25 (55.6)	0.11	63 (57.8)	0.37*
	III non-manual	1302 (24.4)	10 (22.2)		31 (28.4)	
	III manual/IV/V	678 (12.7)	10 (22.2)		15 (13.8)	
Financial difficulties (pregnancy)	None	2312 (42.5)	12 (26.1)	0.08	31 (27.9)	0.02*
	1–3	1712 (31.5)	20 (43.5)		47 (42.3)	
	4 or more	1419 (26.1)	14 (30.4)		33 (29.7)	
Overcrowding (pregnancy)	≤0.5	2751 (50.0)	29 (63.0)	0.17	65 (56.5)	0.72*
	>0.5–0.75	1764 (32.0)	10 (21.7)		26 (22.6)	
	>0.75–1	800 (14.5)	6 (13.0)		19 (16.5)	
	>1	192 (3.5)	1 (2.2)		5 (4.4)	
Car access (pregnancy)	Yes	5293 (95.2)	42 (91.3)	0.22	103 (90.4)	**0.02**
	No	268 (4.8)	4 (8.7)		11 (9.7)	
Private garden/yard (pregnancy)	Yes	4955 (89.3)	38 (82.6)	0.14	94 (93.2)	**0.04**
	No	592 (10.7)	8 (17.4		19 (16.8)	
Damp/condensation/mould in the home (pregnancy)	No	2873 (51.6)	27 (58.7)	0.34	55 (48.3)	0.47
	Yes	2690 (48.4)	19 (41.3)		59 (51.8)	
Postnatal factors						
Financial difficulties (33mo)	None	2043 (39.0)	13 (28.3)	**0.03**	33 (29.0)	0.002*
	1–3	1638 (31.3)	12 (26.1)		31 (27.2)	
	4 or more	1552 (29.7)	21 (45.7)		50 (43.9)	
Housing tenure (at 33mo)	Mortgaged	4489 (85.7)	34 (73.9)	**0.02**	84 (74.3)	**0.001**
	Rented	751 (14.3)	12 (26.1)		29 (25.7)	
Overcrowding (at 33mo)	≤0.5	1065 (20.7)	7 (15.2)	0.45	24 (21.6)	0.44*
	>0.5–0.75	2017 (39.2)	20 (43.5)		40 (36.0)	
	>0.75–1	1754 (34.1)	15 (32.6)		35 (31.5)	
	>1	305 (5.9)	4 (8.7)		12 (10.8)	
Car ownership (at 33mo)	Yes	4916 (94.0)	38 (82.6)	**0.001**	97 (85.8)	**<0.001**
	No	314 (6.0)	8 (17.4)		16 (14.2)	
Private garden/yard (at 33mo)	Yes	4943 (94.5)	43 (93.5)	0.77	103 (91.2)	0.13
	No	290 (5.5)	3 (6.5)		10 (8.9)	
Damp/condensation/mould in the home (at 33mo)	No	2375 (47.1)	22 (50.0)	0.70	45 (40.9)	0.20
	Yes	2672 (52.9)	22 (50.0)		65 (59.1)	

* *p*-value for trend. Significant *p*-values are in bold.

### Factor analyses

For the prenatal factor analysis, the best fit was a single factor model that incorporated all of the SES variables (eigenvalue=1.82; Table 2); the highest factor loadings were observed for maternal education, social class, and paternal education. The best fit for the postnatal factor analysis was also a single-factor model (eigenvalue=1.13), with loadings on housing tenure, private garden/yard use, access to a car, and financial difficulties. All of the variables loaded on the single factor at of 0.2 or above, except for damp, condensation, and mould in the house, which showed minimal loading (<0.15). For the combined-factor model, there was a suggestion of two factors (the first eigenvalue was 2.73 and the second was 1.08), but examination of the scree plot and the loading patterns suggested that a one-factor solution was the most parsimonious choice (Fig. S1, online supporting information).

**Table 2:** Variables and factor loadings for the one-factor model at each time point and combined

Variable	Prenatal factor loading (*n*=3844)	Postnatal factor loading (*n*=5556)	Combined factor loadings (*n*=3403)
Mother’s education	**0.59**	–	**0.46**
Partner’s education	**0.51**	–	**0.43**
Maternal grandmother’s education	0.39	–	0.23
Maternal grandfather’s education	**0.41**	–	0.26
Social class	**0.57**	–	**0.49**
Housing tenure (pregnancy)	**0.45**	–	**0.61**
Overcrowding (pregnancy)	**0.47**	–	**0.52**
Financial difficulties (pregnancy)	0.34	–	**0.43**
Car access (pregnancy)	0.33	–	**0.44**
Private garden/yard (pregnancy)	0.32	–	**0.42**
Damp/condensation/mould (pregnancy)	0.02	–	0.08
Housing tenure (at 33mo)	–	**0.60**	**0.61**
Overcrowding (at 33mo)	–	0.20	**0.40**
Financial difficulties (at 33mo)	–	**0.40**	**0.44**
Car ownership (at 33mo)	–	**0.51**	**0.47**
Private garden/yard (at 33mo)	–	**0.51**	**0.42**
Damp/condensation/mould (at 33mo)	–	0.13	0.08

These factor analyses were restricted to the children in whom data for the demographic variables of interest were available. Factor loadings above 0.4 are in bold.

### Comparison of individual and composite SES measures

The relationship of the prenatal, postnatal, and combined factor scores to Tourette syndrome and Tourette syndrome/chronic tics were examined, along with the four individual SES variables that were the best predictors in the univariable analyses (Table 3). Both the postnatal and combined factor scores, but not the prenatal factor scores, showed positive trends, indicating that worse socio-economic circumstances were associated with Tourette syndrome and Tourette syndrome/chronic tics. However, only the postnatal score was significantly associated with Tourette syndrome, whereas both the postnatal and combined composite scores were associated with Tourette syndrome/chronic tics. Comparison of the different individual and composite variables for goodness of fit identified housing tenure at 33 months as the variable with the best fit (Table SI, online supporting information) for both Tourette syndrome and the Tourette syndrome/chronic tics outcomes. For the factor scores, the postnatal model provided the lowest Akaike information criterion and Bayesian information criterion (best fit) for Tourette syndrome and Tourette syndrome/chronic tics, although the differences between these three derived-factor models were minimal.

**Table 3:** Odds ratio for each standardized factor score and the most relevant individual scores

Factor or variable	Category	Odds ratio for Tourette syndrome (95% CI)^a^	Odds ratio for Tourette syndrome/chronic tics (95% CI)^a^
Prenatal factor score	1	1.00	1.00
	2	0.61 (0.22–1.68)	1.11 (0.61–2.01)
	3	1.63 (0.74–3.60)	1.40 (0.80–2.48)
Postnatal factor score	1	1.00	1.00
	2	1.59 (0.70–3.58)	1.05 (0.61–1.80)
	3	**2.27 (1.06–4.86)**	**2.09 (1.38–3.47)**
Combined factor score	1	1.00	1.00
	2	1.39 (0.48–4.03)	**2.09 (1.04–4.21)**
	3	**2.78 (1.08–7.13)**	**2.78 (1.42–5.43)**
Financial difficulties (pregnancy)	None	1.00	1.00
	1–3	**2.27 (1.10–4.65)**	**2.06 (1.30–3.26)**
	4+	1.64 (0.73–3.66)	1.64 (0.99–2.71)
Car access (pregnancy)	Yes	1.00	1.00
	No	1.42 (0.44–4.62)	**1.93 (1.00–3.74)**
Private garden/yard (pregnancy)	Yes	1.00	1.00
	No	1.82 (0.84–3.93)	**1.72 (1.04–2.84)**
Financial difficulties (33mo)	None	1.00	1.00
	1–3	1.15 (0.53–2.54)	1.14 (0.69–1.87)
	4+	**2.13 (1.07–4.28)**	**2.00 (1.28–3.12)**
Housing tenure (33mo)	Mortgaged	1.00	1.00
	Rented	**2.14 (1.10–4.14)**	**2.09 (1.36–3.21)**
Car ownership (33mo)	Yes	1.00	1.00
	No	**3.34 (1.55–7.22)**	**2.62 (1.52–4.50)**

a Analyses were adjusted for maternal age at the birth of the child and the time-relevant relationship status of the mother. High factor scores relate to low socio-economic status. All the values in bold are when we can refute the null hypothesis as the 95% confidence intervals does not cross the null value of 1. Hence all the *p*-values are by definition less than 0.05.

## Discussion

This prospective cohort study found that a multidimensional composite measure of SES obtained before disease onset predicted the risk of Tourette syndrome and Tourette syndrome/chronic tics. This is the first study to examine this relationship in a large, unselected, prospective population-based cohort using a wide range of SES variables. Lower maternal SES was associated with a twofold increase in the risk of Tourette syndrome and Tourette syndrome/chronic tics in this sample. Mothers of children with a chronic tic disorder at the age of 13 years had experienced significantly more financial difficulties (including lower rates of home and car ownership) during pregnancy and when the child was 2 to 3 years old than mothers of children in the comparison group. Composite measures of SES during the pre- and postnatal periods were also strongly associated with Tourette syndrome/chronic tics. Although the strongest relationship was seen for car ownership at 33 months (odds ratio [OR] 3.3 for Tourette syndrome and 2.6 for Tourette syndrome/chronic tics), the overall SES factor scores, particularly the combined SES factor score, also showed strong associations with Tourette syndrome and Tourette syndrome/chronic tics in childhood, with the greatest risk of the lowest SES tertile (OR 2.8).

Although the postnatal factor variables in our study were generally more strongly associated with Tourette syndrome/chronic tics, the postnatal period may not be of greater aetiological relevance than the prenatal period for Tourette syndrome/chronic tics risk. Postnatal variables also reflect prenatal adversity, as the care of a young child imposes many financial burdens in terms of clothing, feeding, and child care. Thus, parents who were just coping before the birth of the child could be tipped over into financial difficulties in the postnatal period. Similarly, housing tenure may change postnatally, owing to greater space requirements.

We also examined the index of multiple deprivation score, a census-based ecological measure^14^ commonly used in the UK as it does not require individual-level data. We evaluated its utility, as many data sets have limited individual-level SES data but have residential data to enable census linkage. We found no association with Tourette syndrome or Tourette syndrome/chronic tics (data available from authors), suggesting that individual-level SES data are a far more sensitive exposure than an ecological proxy measure in this dataset.

The majority of previous studies that have examined the association between tic disorders and SES found no association.^6^–^8^,^10^,^11^ However, all but two of these studies were conducted in clinical rather than population-based samples. Motlagh et al.^6^ found no association between individuals with Tourette syndrome recruited from a tic disorder clinic and SES using mean years of parental education or mean SES derived using the Hollingshead four-factor index of social status, which examines education, occupation, sex, and marital status.^16^ Whitaker et al.^7^ prospectively assessed maternal social disadvantage before birth and in children with Tourette syndrome at the age of 6 years using composite indexes.^16^ They found no association between tic disorder at 6 years of age and social disadvantage measured at either time point, although the cohort was restricted to children with low birthweight, limiting its generalizability. Klug et al.^8^ also found no evidence of an association between Tourette syndrome and SES assessed via maternal and paternal education in individuals from a disease registry. Similarly, Khalifa et al.^10^ failed to find a cross-sectional association, although this was based on a small sample size (maximum sample size 34 for chronic motor tics) and may reflect a type II error owing to the lack of power. Furthermore, the Khalifa study used occupational class and educational level as measures of SES, which may not have adequately captured social disadvantage. We similarly failed to find statistically significant effects when using these measures alone compared with our multiple-indicator composite score. In contrast, Peterson et al.,^11^ in a population-based study from Upstate New York, did find cross-sectional associations with lower SES and tic disorders, using a composite that included maternal and paternal years of education, paternal occupational status, and family income. We did not find an association between tic disorders and maternal education or occupational social class, although we did find an association between tic disorders and financial difficulties, car ownership, and housing tenure, all of which are correlated with family income, consistent with the findings of Peterson et al.

The discrepant findings between clinic- and population-based studies, including ours, may reflect differences in statistical power and/or biased ascertainment, as all cases in the clinic-based studies were ascertained through specialist services. Since children in the ALSPAC cohort from more affluent families may be more likely to seek medical care,^9^ and thus might be more likely to receive a diagnosis of a tic disorder, one might expect an association of tic disorders with *higher* SES, and thus an association between *lower* SES and Tourette syndrome or Tourette syndrome/chronic tics would be attenuated towards the null. We believe that, for severely affected individuals, there would be little if any differential ascertainment by SES, but for mildly affected individuals, the bias would, if anything, reduce the size of the association.

There are two explanations that are likely to explain the identified association between lower SES and increased risk of Tourette syndrome/chronic tics in this sample. The first is that unidentified environmental risk factors (such as alcohol exposure in pregnancy) are associated with lower SES and are causally linked to Tourette syndrome/chronic tics. Such environmental risk factors may be acting independently or interacting with genetic susceptibility to increase the risk of Tourette syndrome/chronic tics, resulting in an increased expression of symptoms.

Alternatively, the identified association may be the result of psychopathology, either overt or covert, in the mother or father, leading both to increased financial instability (and hence lower SES) and to an increased genetic risk of symptomatology in the child. We cannot differentiate between these two possible explanations for the identified association, as we have no data on parental symptoms, but future aetiological studies of Tourette syndrome/chronic tics should incorporate a measure of SES, both to prevent potential confounding, and to more fully examine the differences in exposure associated with social gradient. Our study also suggests that a composite measure of SES derived from available individual SES variables may be the most robust and, therefore, the most useful measure.

### Strengths and limitations

A major advantage of the ALSPAC study is that it is both population based and prospective, thus avoiding many sources of ascertainment bias often inherent in studies that use participants ascertained from medical services, as well as recall bias which is seen in cross-sectional or retrospective reporting of SES. In ALSPAC, SES data were collected during pregnancy and when the child was 33 months old, long before the onset of tics, avoiding concerns about reverse causation. The primary disadvantage is the reliance on maternal reporting of tic symptoms to assign diagnoses rather than using direct clinical assessments. Given our stringent DSM-IV-based criteria,^13^ we feel it is unlikely that we have overdiagnosed Tourette syndrome or chronic tics; however, as direct clinical assessments often identify additional individuals with mild symptoms that were previously unnoticed by patients or their families, we may have under identified more mildly affected children with Tourette syndrome.^17^ Whilst this would reduce the total number of identified individuals with Tourette syndrome, and hence the power, it is unlikely to have biased our associations with SES, unless mothers from poorer homes were more likely to report mild symptoms than those from more affluent homes. This seems counterintuitive, and studies of adult chronic diseases suggest individuals with lower SES tend to underreport rather than over report symptoms.^18^ Tic disorders in the USA, along with other psychological problems, are also likely to be more severe in publicly insured children than in privately insured children.^19^ This suggests that we have underestimated the true association between tic disorders and SES.

A more significant potential problem is the loss to follow-up bias owing to cohort attrition, as we found that lower SES was associated with a lack of attendance in our study at the age of 13 years. However, if this loss was differential by lower SES and greater risk of Tourette syndrome/chronic tics status, then again, our results would underestimate the true association, unless children from poorer families with Tourette syndrome/chronic tics were more likely to remain in the study. This study sampled children from the UK, so one should be cautious in generalizing to other populations.

The association between social inequalities and child health is not unique to Tourette syndrome/chronic tics. A recent systematic review and meta-analysis highlighted worse outcomes in relation to birthweight, preterm birth, and infant, neonatal, and postneonatal mortality in infants with lower SES in the UK.^20^ Similarly, a large US study in California found that the risk of cerebral palsy among 6.2 million live births showed a marked dose–response relationship with years of maternal education (33% greater risk among females with only primary school education compared with college graduates), although this effect was seen among white, Hispanic, and Asian (but not black) mothers.^21^ There is conflicting evidence for an association between SES and autism spectrum disorders, but many studies have shown a greater risk in more affluent populations. The large Autism and Developmental Disabilities Monitoring Network found a twofold (2.08) prevalence ratio comparing high-SES with low-SES areas, but only a 40% relative increase (1.40) when comparing children without a pre-existing diagnosis.^22^ This elegantly demonstrates the same issue of ascertainment bias that is likely to occur in the Tourette syndrome/chronic tics literature.

These results should encourage other researchers to measure and understand the reasons for the social patterning of Tourette syndrome and chronic tics as well as other childhood neurodevelopmental disorders. We recommend using multiple measures of SES, though the specific choice of variables will depend on the cultural significance of each variable and will differ by country and secular period. Our ecological measure of area deprivation did not show any real association, suggesting that this may be insensitive as a marker of disease risk. This does not exclude the potential role of geographically-based toxic environmental factors, as we did not examine geographical clustering, and our study area may not have been sufficiently heterogeneous with regard to these exposures. Future work will explore which of the many individual risk factors measured in the ALSPAC cohort could explain these social differences in disease risk in the hope that this would highlight potentially modifiable factors that could result in the primary prevention of Tourette syndrome and chronic tics.

## Abbreviations

ALSPAC: Avon Longitudinal Study of Parents and Children
SES: Socio-economic status

## SUPPORTING INFORMATION

The following additional material may be found online:

Appendix S1 Further details on how the SES variables were coded.

Table SI Akaike information criterion (AIC)/Bayesian information criterion (BIC) (goodness of fit) statistics.

Figure S1 Scree plots for the prenatal, postnatal, and combined SES factor models.
