# Supplementary material for: Tourette syndrome and chronic tic disorder are associated with lower socio-economic status: findings from the Avon Longitudinal Study of Parents and Children cohort
Source: Dev Med Child Neurol. 2013 Oct 19;56(2):157–63. doi: 10.1111/dmcn.12318 (PMC3908357; doi:10.1111/dmcn.12318)
Supplement: Appendix S1 — Further details on how the SES variables were coded. [file dmcn0056-0157-sd1.docx]

Appendix 1: Further details on how the SES variables were coded

Educational level was based on the highest reported educational achievement for the subject’s mother, her partner and maternal grandparents. Data on the partner was taken from either a partner questionnaire or maternal report if missing. Education was classified on the age at which formal education was completed (< 16 years old, 16 years (reference group), or > 16 years). Occupation-based social class was identified from the maternal questionnaire collected at 32 weeks gestation for the mother and her partner. Details of current or most recent employment were used to categorize social class into an ordinal variable based on highest value from both parents: social class I (e.g. professionals such as doctors), II (managerial or technical), IIInm (skilled non-manual), IIIm (skilled manual), IV (partly-skilled), and V (unskilled, e.g. laborers). We then grouped this into tertiles (I/II, IIInm, and IIIm/IV/V), based on the frequency distributions. Housing tenure was dichotomized as mortgaged/owned vs. rented/other and assessed at 18 weeks gestation and when the child was 2-3 years old. Overcrowding was calculated as the number of people per room (categorized as <=0.5, >0.5-0.75, >0.75-1, >1) both during pregnancy and when the child was 33 months of age. Financial difficulties during pregnancy and at 33 months were established by asking how difficult the mother found it to afford five essential purchases (food, clothing, heating, accommodation and items for the baby: total score ranged from 0 to 15). This was categorized into 0 or ‘no financial difficulties’, 1-3, or ‘some financial difficulties’, and 4-15, ‘definite financial difficulties’. Dichotomized variables (yes/no) were created for car ownership, sole access to a garden/yard, and presence of damp, condensation, or mould in the home based on the 33 month maternal questionnaire.
