# Supplementary material for: Tourette syndrome and chronic tic disorder are associated with lower socio-economic status: findings from the Avon Longitudinal Study of Parents and Children cohort
Source: Dev Med Child Neurol. 2013 Oct 19;56(2):157–63. doi: 10.1111/dmcn.12318 (PMC3908357; doi:10.1111/dmcn.12318)
Supplement: Table SI — Akaike information criterion (AIC)/Bayesian information criterion (BIC) (goodness of fit) statistics. [file dmcn0056-0157-sd2.docx]

**Table SI:** Akaike information criterion (AIC)/Bayesian information criterion (BIC) (goodness of fit) statistics.

|  | TS (n=3,030) | | TS/CT (n=3,068) | |
| --- | --- | --- | --- | --- |
| Factor or variable | AIC* | BIC* | AIC | BIC |
| Prenatal factor score | 340.61 | 370.69 | **659.33** | 689.47 |
| Postnatal factor score | **338.16** | **368.28** | 659.46 | 689.64 |
| Combined factor score | 341.82 | 377.92 | 663.22 | **689.39** |
| Financial difficulties (pregnancy) | 339.79 | 369.91 | 662.23 | 692.41 |
| Car access (pregnancy) | 343.51 | 367.57 | **656.31** | **680.43** |
| Private garden/yard | 342.78 | 366.85 | 657.68 | 681.80 |
| Financial difficulties (33m) | 341.21 | 371.33 | 659.42 | 689.60 |
| Housing tenure (33m) | **339.41** | **363.51** | 657.07 | 681.22 |
| Car ownership (33m) | 341.82 | 365.92 | 663.47 | 687.62 |

* AIC and BIC were used to assess goodness of fit. The lowest AIC/BIC of the factor scores and the individual scores are both highlighted. Variables that predicted either Tourette syndrome or Tourette syndrome/chronic tic (Table 1) were included in this stage of modelling. Note that sample sizes are lower than for assessment of individual variables because we have used the complete case analysis to ensure comparability
