# Supplementary material for: Tourette syndrome and chronic tic disorder are associated with lower socio-economic status: findings from the Avon Longitudinal Study of Parents and Children cohort
Source: Dev Med Child Neurol. 2013 Oct 19;56(2):157–63. doi: 10.1111/dmcn.12318 (PMC3908357; doi:10.1111/dmcn.12318)
Supplement: Figure S1 — Scree plots for the prenatal, postnatal, and combined SES factor models. [file dmcn0056-0157-sd3.docx]

e-figure: Scree plots for the prenatal, postnatal and combined SES factor models.
